# The patient with rhinitis in the pharmacy. A cross-sectional study in real life

**DOI:** 10.1186/s40733-015-0002-6

**Published:** 2015-06-04

**Authors:** Carlo Lombardi, Eleonora Musicco, Francesco Rastrelli, Germano Bettoncelli, Giovanni Passalacqua, Giorgio Walter Canonica

**Affiliations:** 1 Unit of Allergy-Clinical Immunology & Respiratory Diseases - Department of Medicine & Geriatrics, Poliambulanza Hospital Institute, Brescia, Italy; 2 President of the Order of Pharmacists of Brescia, Brescia, Italy; 3 General Practitioner, Brescia, National Responsible for the Pulmonology Area of the Italian Society of General Practitioners (SIMMG), Brescia, Italy; 4 Allergy & Respiratory Diseases, Department of Internal Medicine, University of Genoa, Genoa, Italy

**Keywords:** Respiratory allergy, Allergic rhinitis, Allergic asthma, Pharmacist

## Abstract

**Background:**

In the practical management of allergic rhinitis (AR), pharmacists are usually the first-line contact, also because some medications are available as over the counter. Therefore, pharmacists may represent an important resource, in mediating the interaction between patients and physicians. We evaluated the clinical/demographic characteristics of patients with respiratory allergies who consulted their pharmacists as first-line contact. A patient-oriented questionnaire was developed by a scientific committee including pharmacists, GPs, allergists, pulmonologists and ENT specialists.

**Methods:**

The questionnaire consisted of items covering the general aspects of AR. Allergic Rhinitis and its Impact on Asthma guidelines were assumed as reference for diagnosis and therapy. The questionnaire was distributed to pharmacies, and pharmacists were asked to deliver the questionnaire to all patients referring for nasal symptoms.

**Results:**

30 pharmacies were involved during the pollen season 2011, and 410 patients (55 % male) participated. The most frequent complaints were 20 rhinitis (49 %) and conjunctivitis (29 %), followed by lower respiratory symptoms (cough and/or dyspnea). Isolated conjunctival symptoms were present in only 22 % of patients. Among patients with lower respiratory symptoms, cough was the most frequent, variously associated with upper respiratory symptoms or overt dyspnea. Dyspnea alone was present in 16 % of patients. 39 % of patients had no physician-based diagnosis. Oral antihistamines were the most used self-medication, followed by intranasal decongestants. 30 % of respondents had used alternative medicines.

**Conclusion:**

According to these data, AR is still considered a trivial disease, frequently self-managed, with over the counter medications, not in line with guidelines. A physician-based diagnosis is present in about 60 % of patients.

## Background

Respiratory allergic diseases (asthma/rhinitis) probably represent in absolute the most frequent immune-mediated disorders [1], and their prevalence is still increasing worldwide, as recently underlined in the GEIRD (Gene Environment Interactions in Respiratory Diseases) epidemiological study [2]. The impact of respiratory allergy is of particular relevance when considering the costs, either direct (expenditure for drugs, hospitalizations, access to medical care) or indirect (absenteeism, presenteism, decreased school/work performance). Allergic Rhinitis (AR) is defined as a symptomatic disorder of the nasal mucosa, due to an IgE-mediated inflammation that follows the contact with an offending allergen. Cardinal symptoms of AR are nasal itching, sneezing, rhinorrhea and obstruction, and they are spontaneously reversible or controlled by adequate treatment [3–5]. AR can be frequently associated with other comorbidities, such as rhinosinusitis which is present in about 20–30 % patients [6], asthma (10–35 %) [7], and conjunctivitis. Also polyposis and sleep disturbances [8,9], are not rare, occurring in not less than 10 % of subjects. This aspects make the diagnostic approach sometimes particularly complex [10]. Nonetheless, AR is still considered as a “trivial” disease, that can be easily managed by the patient himself or by healthcare providers other than physicians. In this context, pharmacists are usually the first-line contact for AR patients [3,11], also because, several medications (namely, oral/local antihistamines and decongestants) are available as “over the counter” [12]. Therefore, pharmacists with an adequate preparation may represent an important healthcare resource, in mediating the contact between patients and physicians. This is especially true when a more detailed diagnostic approach is needed. Based on these considerations, we aimed at evaluating the clinical/demographic characteristics of patients suffering with respiratory allergies who consulted pharmacists as first-line contact. This was done by a questionnaire-based survey.

## Methods

A patient-oriented questionnaire was developed by a scientific committee in which convened pharmacists (25 %), GPs (20 %), allergists (25 %), pulmunologists (15 %) and ENT (15 %) specialists. The questionnaire consisted of 12 items covering the main and general aspects of AR, as shown in Table 1. The questionnaire was discussed and agreed by the Provincial Board of Pharmacists of Brescia.

**Table 1:** Template of the questionnaire used

	**QUESTION**	**POSSIBLE ANSWERS**
1	Gender	• M
		• F
2	Age (years)	• <20
		• 21-40
		• 41-60
		• >60
3	Race	• Caucasian
		• Asian
		• South American
		• Arab
		• Other………….
4	Which symptom mainly prompted you to go to the pharmacy?	• Rhinitis (sneezing, runny nose, itchy nose, stuffy nose)
		• Cough
		• Conjunctivitis (burning eyes, itchy eyes, photophobia)
		• Dyspnea (short breath)
5	How long before your visit to the pharmacy did the symptom appear?	• < 5 days
		• 5-10 days
		• 11-30 days
		• > 30 days
6	The problem was already diagnosed by a physician?	• Yes
		• No
7	Did you received a physician prescription for your symptoms?	• Yes
		• No
8	Which medications do you usually take? (multiple answers allowed)	• Topical nasal decongestants
		• Systemic antihistamines
		• Topical antihistamines
		• Topical steroids
		• Systemic steroids
		• Topical anticholinergics
		• Topical Cromones
		• Antibiotics
		• Antileukotrienes
		• If you can not specify the class, indicate commercial names
9	Do you use or have used complementary/alternative medicines (e.g. homeopathy, acupuncture, herbs) for your rhinitis/asthma?	• Yes
		• No
10	Do you use or have used allergy vaccines?	• Yes
		• No
		If yes:
		• Sublingual route
		• Subcutaneous route
11	If for this problem you usually care alone, why do you?	• It is a trivial problem
		• It’s not worth to talk to the doctor
		• The doctor underestimates
		• Just consult the pharmacist
12	Do your symptoms affect your everyday life?	• Not at all
		• Moderately
		• Heavily

The Allergic Rhinitis and its Impact on Asthma (ARIA) document was assumed as guideline for diagnostic and therapeutic approaches [3,10]. The questionnaire, as a paper sheet, was distributed to pharmacies of the Brescia province and pharmacists were asked to deliver the questionnaire to all patients referring for nasal symptoms during the 2011 pollen season (February –May). The participation into this survey was on a voluntary basis and all the personal/demographic data were kept strictly anonymous. The observational study was notified to the Conjunct Commission of Brescia Phisicians & Pharmacists Orders, according to the Italian legislation.

## Results

**Fig. 1:**
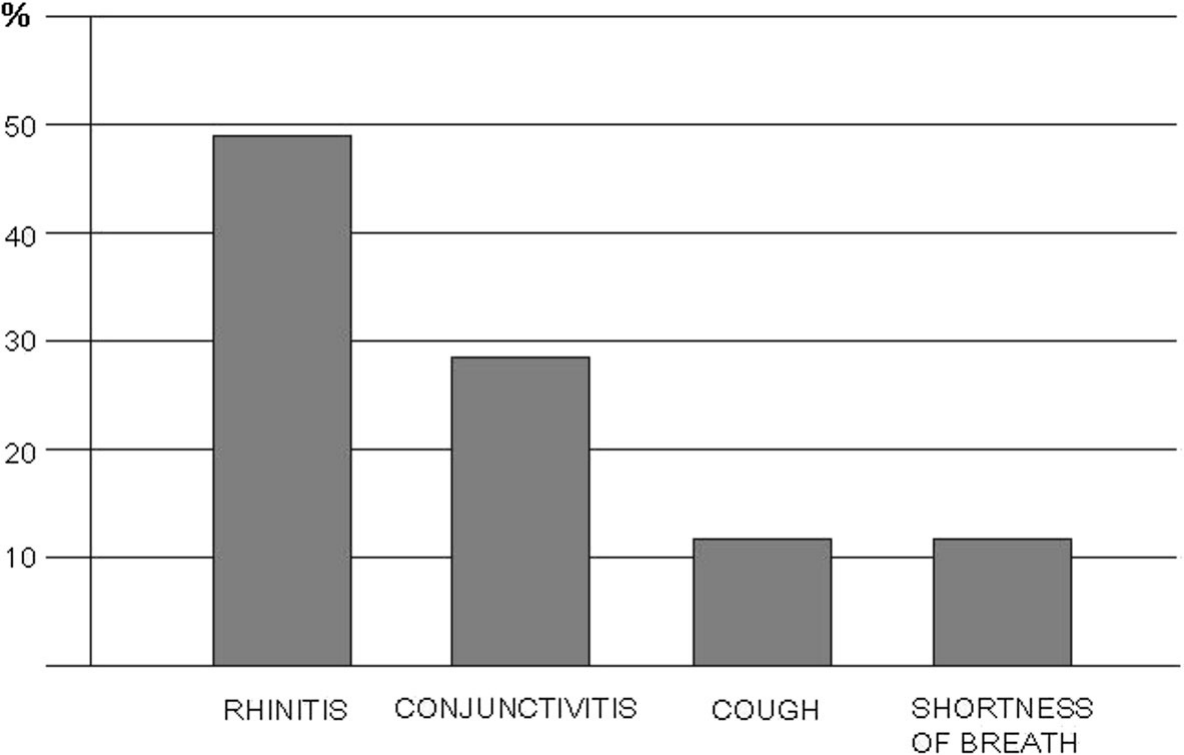
Main Symptom reported by patients as reason for referral to pharmacy

**Fig. 2:**
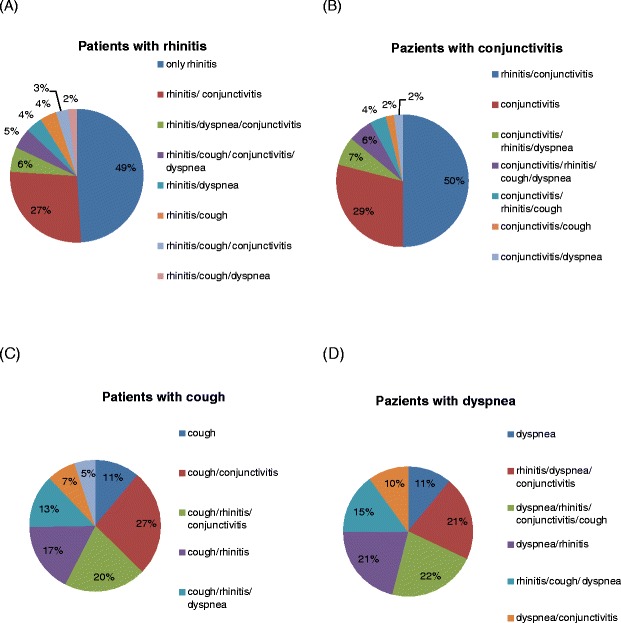
The main symptom that leaded patients to pharmacies is confirmed to be rhinitis **(a)**. Conjunctivitis **(b)**, cough **(c)**, and dyspnea **(d)**, often associated with other allergic symptoms, in particular rhinitis

**Fig. 3:**
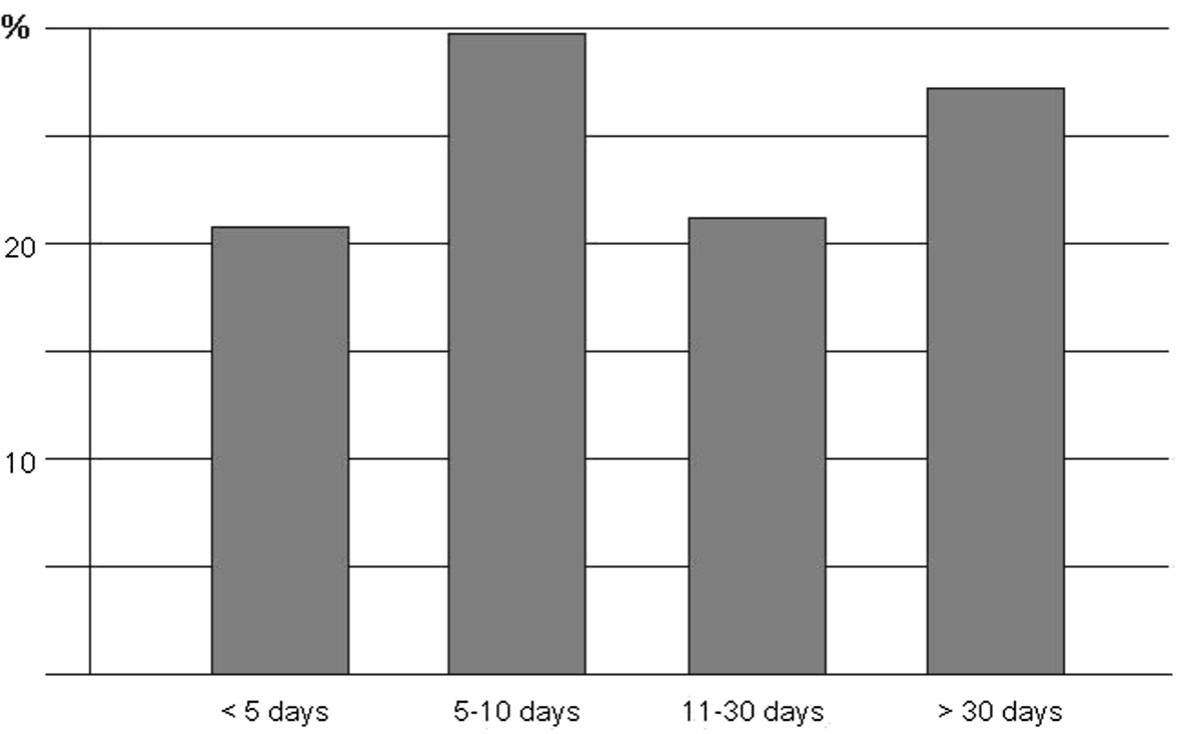
Latency time between the onset of symptoms and the access to pharmacy. There is a wide distribution in the time period considered

**Fig. 4:**
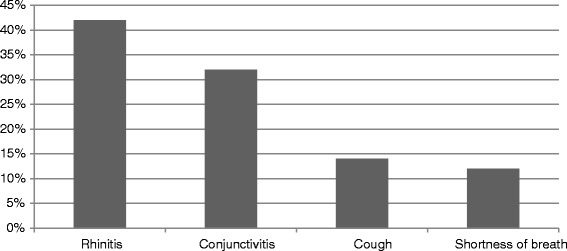
Even among immigrant patients rhinitis remains the predominant allergic disease leading patients to the attention of the pharmacist

**Fig. 5:**
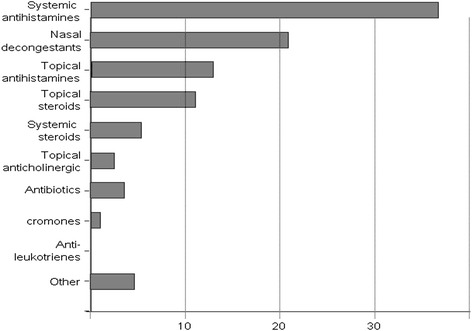
Drug treatment mainly used/required by patients referred to the pharmacy for respiratory allergies. Antihistamines (36 %) and topical nasal decongestants (21 %) predominate

Thirty pharmacies, 54 % in the urban area of Brescia and 46 % in the surroundings, participated in this initiative. At the final collection, there were 410 respondent patients (55 % male, age range: 20–40 years), all referring to pharmacies during the spring pollen season 2011 (February–May) for nasal/respiratory complaints (rhinitis, rhinosinusitis, asthma, conjunctivitis). Of note, the majority of subjects belonged to an age range characterized by active work/school/sport activity. Out of the 410 patients, 19 % were immigrants (4 % african, 3 % arab, 3 % asian, 2 % latin american, 7 % eastern european). This is in line to what previously observed in a cross-sectional study performed in this geographical area [13]. According to questionnaires, the most frequent complaints were rhinitis (49 %) and conjunctivitis (29 %), followed by lower respiratory symptoms (cough and/or dyspnea) (Fig. 1). Rhinitis symptoms remained the main cause of spontaneous access to the pharmacy (320 patients out of 410 total, 76 % of patients with rhinitis), responsible alone of 49 % of consultations, and associated with conjunctivitis in 27 % of the accesses (Fig. 2a). Isolated conjunctival symptoms did not appear as a relevant event, accounting for 29 % of patients (Fig. 2b). Among patients with lower respiratory symptoms, cough was the preeminent reason for referral, and it was variously associated with upper respiratory symptoms or overt dyspnea (Fig. 2c). On the other hand, dyspnea alone was reported by 11 % of patients (Fig. 2d) . There was a wide time span between the onset of symptoms and the seek for care (Fig. 3), and in 39 % of patients no physician-based diagnosis was declared, this underlining a high proportion of self-diagnosis and self-management. Notably, in 49 % of immigrants, the problem had been not formally diagnosed, and 81 % were not treated by a doctor. These patients were therefore underdiagnosed and undertreated. Even among immigrants, rhinitis was the disease that led patients to the attention of the pharmacist (Fig. 4). In general, Oral antihistamines (over the counter in Italy) were the most used self-medication drug, followed by intranasal decongestants (self medication in 22 % of patients) (Fig. 5). Treatment options such as homeopathy or alternative medicine had already been used or actually used by 33 % of the respondents. Concerning allergen-specific immunotherapy sublingual or subcutaneous, only 12 % of the patients examined in our study had used it. The pharmacist resulted to be the first-line contact for 47 % of patients, whereas 42 % of the respondents considered its problem as trivial, and 11 % reported that GPs under evaluated nasal symptoms. Finally, 28 % of the respondents considered AR as heavily impacting on the quality of life and another 59 % judged the impact as moderate.

## Discussion

AR is a common and frequent healthcare problem, affecting almost 25 % of the general population [1,2]. The epidemiological impact lead to the search for a better diagnostic definition and for an evidence-based approach to its therapy, as done with the ARIA guidelines [10], also considering that patients’ profile is becoming more and more complex [14]. Nonetheless, AR still remains considered a trivial disease, since it is not life-threatening and easy to manage [15]. For those reasons AR is frequently self-managed by patients, and the pharmacist, therefore, represent the first-line contact. Guidelines dedicated to pharmacists have been already published [4], but their real impact on everydays’ practice have been not been specifically addressed so far. Thus, we attempted to evaluate the characteristics, diagnosis, treatment profile and perception of AR in a pharmacy setting. As expected, a vast majority of patients still retain the use of “over the counter” medicines as the primary resource, being rhinitis a self-manageable disease. Also, in line with the internationally increasing use of Complementary-Alternative Medicines [16], according to Italian data about one third of patients approached these therapies for their diseases or symptoms [17]. In general it is known that the reasons for the choice of the use of complementary medicines are multiple, among which prevails the conviction of the lower level of toxicity compared to conventional treatments [18].

## Conclusion

According to our data, collected by pharmacists, a large percentage of patients still consider AR as a trivial disease [19], and recur to self-administered drugs (oral/nasal antihistamines and/or nasal vasoconstrictors), whereas an appropriate diagnosis is made only in few cases. In particular, the use of intranasal decongestants testifies for a non-optimal management of AR, as well as the poor referral to physicians. This is in line with elsewhere reported data based on pharmacists’ experience [20–22]. Finally, the use and knowledge of allergen-specific immunotherapy seems to need improvement. Based on the present results, a more strict and bi-directional collaboration between pharmacists and physicians would be auspicable.
